# Items of General Interest

**Published:** 1916-07

**Authors:** 


### Items of General Interest.

### Attorney General Rules in Favor of the Rural Districts.

A recent ruling of the Attorney General's department is of special interest to the unincorporated towns and rural districts. The Revised Statutes provide that the commissioners court shall have the power to divide the unincorporated portions of the county into health districts, may appoint a board of health for this district and provide improvements necessary and pay for same.

The International Health Commission and the University of Texas, in conjunction with the State Board of Health, have instituted a campaign in the rural districts of Leon county. In this campaign, three districts are selected, every person is examined for hookworm, typhoid and malaria; those found to be infected are treated; with the assistance of the householder, sanitary privies are built, houses are screened and disease-breeding places are rendered sanitary.

Harris county has recently authorized a campaign to cover the entire portion of the county not included in the incorporated cities and towns. During this campaign, a board of health for each district will be selected. This board will hold the same relation to the district that the city board of health does to the city. In this way, the people of the rural districts will have the protection to which they are entitled.

In discussing the power of the commissioners court to appropriate the county funds for this purpose, the Attorney General's department says that the commissioners court is authorized to make such appropriation and that the character of work now being done seems to be in exact accord with and of the character contemplated should be done by this statute.

Until recently, authorities have believed that the cities furnished the rural districts with their disease; that the country home was safer from a health standpoint than the city home. Statistics show the opposite to be true.

The division of the rural portions of a county into health districts, appointment of a board of health, the eradication of hookworm, typhoid fever and malaria, will show marked effects, not only on the health of the people, but also their prosperity.

---

Parkview Retreat, Greenville, Texas, was closed April 15th for the purpose of enlarging its capacity to a thirty-room institution. It was reopened July 1st.

---

According to the Philadelphia press the Pasteur Institute of Paris has just made the declaration that one of the most powerful stimulants known is milk. It has been used for months as the one stimulant for the French soldiers in the trenches.

---

One medical outcome of the war is the discovery that sphagnum moss and fine sawdust are excellent substitutes for absorbent cotton as dressings for wounds. The moss is the long-fibered kind commonly found in swampy places in this country that florists and nurserymen use for protecting the roots of plants during shipment.

---

If we supinely submit to legislation detrimental to our best interests, without protesting, we have no moral right to complain about our laws and lawmakers.—*W. F. Zierath, Wisconsin Medical Journal.*

---

### The Truth About Tuberculosis.

It was unanimously agreed by the physicians of the medical section of the Southern Medical Association in session for the discussion of tuberculosis that the essential and most urgent need in dealing with the white plague is the making of early diagnosis even before the physical symptoms become at all evident. If the most prevalent of all diseases—tuberculosis—is to be successfully combated and the human race saved from the hundreds of thousands of deaths that occur annually from this disease, the general practitioner must learn how to make these early diagnoses and must make them and give their patients the benefit of treatment before any of the physical signs become evident. If treatment is administered in the very first stages, it was declared, the patient will be permanently cured; but if treatment is delayed until the advanced stages are reached, cure is next to impossible, the patient becomes an invalid for life and eventually succumbs to the ravages of the disease.

The symposium on tuberculosis was opened by Dr. S. E. Thompson of the Texas Tuberculosis Sanatorium, Carlsbad, Texas, treating the subject, "Common Sense and the Fever Thermometer Versus the Microscope in the Early Diagnosis of Pulmonary Tuberculosis."

### SOME SERIOUS THINGS.

"It is a serious thing," Dr. Thompson said, "to tell a patient that he has tuberculosis. But it is a much more serious thing to delay the diagnosis and therefore the treatment in the early stages. The need for more emphasis on this point is made evident by the fact that not more than 2 per cent of tuberculosis patients are in the incipient stage when they reach our sanatoriums. Ninety-eight per cent of general practitioners do not know how to make the diagnosis for pulmonary tuberculosis because there are no physical signs and the diagnosis is an extremely difficult one. And of the cases sent to the sanatoria a discouraging low percentages described by the general practitioners sending them as being in the incipient stage are really in the advanced stages and therefore incurable.

"The fault is in our system of teaching. Medical students are not taught to make the diagnosis for the early stages, and consequently general practitioners see no indications of tuberculosis until the patient begins to show well-known physical signs which only come in the advanced stages.

### COMMON SENSE SYMPTOMS.

"There are common sense symptoms for which we must look. Waiting for positive sputum is bad, and the diagnosis must be made before germs appear in the sputum if the patient is to be cured. These are some of the symptoms that we must look for if we are to discover the disease in its incipient stage: (1) Fatigue, an unreasonable tired feeling without sufficient exercise to produce it; (2) feeling of being run down; (3) loss of appetite; (4) loss of energy; (5) subnormal morning temperature with a rise above normal during the day; (6) slight morning cough;

(7) chest pains; (8) hoarseness, and (9) rapid pulse above 80. All hemorrhages should be considered as symptoms of pulmonary tuberculosis until proven to be otherwise."

The discussion following Dr. Thompson's paper still more emphatically urged that general practitioners learn the symptoms of tuberculosis and be constantly on the lookout for incipient cases. It was pointed out that 97 per cent of the human race are infected with tuberculosis germs to some extent. These may be successfully combated by the body and never gain a firm hold. On the other hand, breakdowns of the system, from overwork, exposure, or many other causes, may enable these germs to gain a hold. The medical profession should be educated to watch for and recognize these very first indications, and the public should be educated to the importance of clean and decent living so as to keep the body constantly in a physical condition to withstand the tubercular infection to which it is almost constantly exposed.

### REST AND EXERCISE.

Dr. Thompson Frazer, of Asheville, N. C., presented a paper on "Rest and Exercise in Tuberculosis." Dr. Frazer said that neither rest or exercise, the one exclusive of the other, is of any value. They must be employed together, with great care and discretion. Patients suffering from consumption, who are in a weak and broken-down condition, should be put to bed and given a complete rest, he said. After they are well on the way to recovery they should be given a little exercise to begin with, such as walking, and that exercise gradually increased as they grow stronger, until they are in a physical condition to do more work than their regular duties require them to do before they are permitted to return to work. This latter, he said, is of utmost importance. If patients are permitted to return to work before they are wholly able to exert the amount of energy required in that work, relapses are sure to occur.

Dr. Mary Lapham, Highlands, N. C., gave a very interesting talk on "Bronchial Gland Tuberculosis." Dr. Lapham said that the bronchial glands are the source of infection in most all cases of pulmonary tuberculosis. The tubercular germs gain admission to the glands, often in the early childhood of the victim. They may remain dormant there until maturity, or even until late in life, and then, when the body is run down and unable to put up a strong resistance, they may be swept into the lungs and cause pulmonary tuberculosis. There are absolutely no symptoms of tubercular germs in the bronchial glands, Dr. Lapham said. The only means of discovering such an infection in the glands is by an X-ray examination.

Dr. Silvio von Ruck, of Asheville, N. C., read a highly technical paper on "The Recognition and Treatment of Occult Tuberculosis."

OCCULT TUBERCULOSIS.

"Occult tuberculosis," he said, "is a disease about which almost nothing is known, and any disease that does not permit of definite analysis may be suspected to be of tubercular origin. Occult tuberculosis is present in many cases where we do not detect it because the symptoms are covered up by other complications."

Dr. McBrayer, of Asheville. N. C., gave an interesting account of a tuberculosis survey which he had superintended among the silk mill workers of the village of Waynesboro, N. C. The death rate in that village from consumption he found to be at the ratio of sixteen to one above the normal death rate per unit of population for this disease. He told of the methods employed in making the survey, illustrating with charts and maps, and of some of the precautions and remedies recommended for reducing the prevalency of the disease among the workers.

"The Application of Artificial Pneumothorax in the Treatment of Pulmonary Tuberculosis" was the subject of a paper by Dr. Martin F. Sloan, of Towson, Md. Dr. Sloan, who is a well known authority on the pneumothorax method, recommended it not as a cure, but as a treatment for advanced chronic cases for temporary and in some cases, practically permanent relief where conditions are right. The remedy, he said, is only for such cases as are beyond cure, and the greatest of care should be used in selecting such cases. In fact, careful selection is essential if the method is to bring results. Other important things to be considered are the different technique of administering the treatment and the question of subsequent inflations, he said.

